# Black tea prevents cigarette smoke-induced apoptosis and lung damage

**DOI:** 10.1186/1476-9255-4-3

**Published:** 2007-02-14

**Authors:** Shuvojit Banerjee, Palas Maity, Subhendu Mukherjee, Alok K Sil, Koustubh Panda, Dhrubajyoti Chattopadhyay, Indu B Chatterjee

**Affiliations:** 1 Dr. B. C. Guha Centre for Genetic Engineering & Biotechnology, University College of Science, Kolkata 700019, India

## Abstract

**Background:**

Cigarette smoking is a major cause of lung damage. One prominent deleterious effect of cigarette smoke is oxidative stress. Oxidative stress may lead to apoptosis and lung injury. Since black tea has antioxidant property, we examined the preventive effect of black tea on cigarette smoke-induced oxidative damage, apoptosis and lung injury in a guinea pig model.

**Methods:**

Guinea pigs were subjected to cigarette smoke exposure from five cigarettes (two puffs/cigarette) per guinea pig/day for seven days and given water or black tea to drink. Sham control guinea pigs were exposed to air instead of cigarette smoke. Lung damage, as evidenced by inflammation and increased air space, was assessed by histology and morphometric analysis. Protein oxidation was measured through oxyblot analysis of dinitrophenylhydrazone derivatives of the protein carbonyls of the oxidized proteins. Apoptosis was evidenced by the fragmentation of DNA using TUNEL assay, activation of caspase 3, phosphorylation of p53 as well as over-expression of Bax by immunoblot analyses.

**Results:**

Cigarette smoke exposure to a guinea pig model caused lung damage. It appeared that oxidative stress was the initial event, which was followed by inflammation, apoptosis and lung injury. All these pathophysiological events were prevented when the cigarette smoke-exposed guinea pigs were given black tea infusion as the drink instead of water.

**Conclusion:**

Cigarette smoke exposure to a guinea pig model causes oxidative damage, inflammation, apoptosis and lung injury that are prevented by supplementation of black tea.

## Background

Cigarette smoking is a major cause for the increased incidence of Chronic Obstructive Pulmonary Diseases (COPD), worldwide. The pathogenesis of this disease is usually characterized by abnormal enlargement of airspaces of the lung accompanied by destruction of its walls [1]. This is a major and increasing global health problem, which is currently the 4^th ^leading cause of death, and is projected to become the 3^rd ^commonest cause of death and the 5^th ^commonest cause of disability in the world by the year 2020 [2]. However, the cellular and molecular mechanism of COPD is not clear and there are no effective drug therapies for such lung damage that are able to significantly reduce disease progression.

Over the last few decades, inflammation and protease/antiprotease imbalance have been proposed to act as downstream effectors of the lung destruction following chronic cigarette smoking [3]. It is now recognized that alveolar cell apoptosis is a major step in such damage process [1,4-8]. It has been shown that chronic exposure of rats to mainstream cigarette smoke (CS) produces significant and time-dependent increase in the proportion of apoptotic cells in the bronchial and bronchiolar epithelium [9] and also of alveolar macrophages [10]. However there is conflicting evidence for the induction of apoptosis. It is reported that exposure of airway epithelial cells to CS does not cause apoptosis but induces cell death by necrosis only [11]. Notably, one prominent deleterious effect of CS is oxidative damage [12-15]. It is also reported that CS-induced oxidative stress is associated with apoptosis of human lung fibroblasts [16] as well as epithelial cells *in vitro *[17]. In fact, it is now becoming progressively apparent that interactions among oxidative stress, apoptosis and excessive proteolytic damage of the alveolar cells may be responsible for the pathogenesis of cigarette smoke-induced lung damage [1]. However, the question that remains to be addressed is whether oxidative damage precedes apoptosis or vice versa. Earlier we had shown that CS contains some stable water-soluble oxidant that causes significant oxidative damage to microsomal proteins and increased proteolysis [14,15]. Altogether, these results would indicate that CS-induced oxidative damage and proteolysis may lead to apoptosis of alveolar cells and overall damage to the lung, which is likely to be prevented by antioxidants. In fact, oxidative stress has been implicated in the pathogenesis of lung damage as is seen in diseases like emphysema [1], and antioxidant therapy is considered to be a logical therapeutic approach in COPD [18]. Black tea has strong antioxidant properties and this is well vindicated in our previous report which demonstrates that CS-induced oxidative damage of guinea pig lung microsomal proteins and increased proteolysis are markedly prevented by BT [19]. In this paper we demonstrate that the initial event of exposure of guinea pigs to CS is oxidative damage, which is accompanied by inflammation, apoptosis and increased air space in the lung and that all these pathophysiological events are prevented when the CS-exposed guinea pigs are given black tea infusion as the drink instead of water.

## Materials and methods

### Chemicals and reagents

The source of black (CTC) tea was West Bengal Tea Development, Kolkata, India. Antibodies against p53, phosphorylated p53, Bax, Bcl-2, caspase 3 and anti-mouse-HRP, anti-rabbit HRP antibodies as well as the chemiluminescent kit for immunoblot analysis were obtained from Cell signaling Technology, Inc. USA. Anti-tubulin antibody was obtained from Santa Cruz Biotechnology, Inc. The *in situ *cell death detection kit was obtained from Roche. USA. Kit for protein estimation was obtained from Bio-Rad. BCIP/NBT (5-bromo-4-chloro-3-indolyl phosphate/nitro blue tetrazolium) was obtained from Bangalore Genei (India). All other chemicals were of analytical grade.

### Preparation of tea infusion

Tea infusion was prepared as described as before [19]. Two grams of black tea were added to 100 ml of boiling water, brewed for 5 min, cooled to room temperature and filtered. The filtrate has been designated as BT. The sample of black tea (CTC) used contained approximately 1% theaflavins (TF), 18% thearubigins (TR) and 6% catechins (CT) [19].

### Exposure of guinea pigs to Cigarette Smoke (CS)

Male short hair guinea pigs weighing 350–450 g were used for all experiments. All animal treatment procedures met the NIH guidelines [20] and Institutional Animal Ethics committee guidelines. The guinea pig was used as a model animal, because, like humans, guinea pigs cannot synthesize ascorbic acid [21,22]. The guinea pigs were fed an ascorbate-free diet for 7 days to minimize the ascorbate level of plasma and tissues [15]. This is because ascorbate is a potential inhibitor of CS-induced oxidative damage of proteins [14,15], which would otherwise counteract the damaging effect of CS. The diet given to the guinea pigs was similar to that described before [19], except that wheat flour was replaced by wheat bran. After 7 days of vitamin C deprivation, the guinea pigs were given oral supplementation of 1 mg vitamin C/day to prevent onset of scurvy. It is known that a dose of 0.8 mg vitamin C/day is adequate to maintain the guinea pigs [23].

After consuming the ascorbate-free diet for 7 days followed by supplementation of 1 mg vitamin C/animal/day, the guinea pigs were subjected to cigarette smoke exposure from 5 cigarettes/animal/day in a smoke chamber. An Indian commercial filter-tipped cigarette (74 mm) with a tar content of 15 mg and nicotine content of 1 mg was used. The smoke chamber was similar to that of a vacuum desiccator with an open tube at the top and a side tube fitted with a stop cock. The volume of the chamber was 5 litre. The cigarette placed at the top was lit and CS was introduced into the chamber containing the guinea pig by applying a mild suction of 4 cm water through the side tube for 10 sec. After then the vacuum was turned off and the guinea pig was further exposed to the smoke for another 30 sec. The total duration of exposure to smoke form one puff was thus 40 sec. The amount of suspended particle per puff was 2.3 mg. Altogether 2 puffs per cigarette was given, allowing the animal 1 min rest in smoke-free atmosphere to breathe air between each puff. The gap between one cigarette and the next was 1 hour. Pair-fed sham controls were subjected to air exposure instead of CS under similar conditions.

The guinea pigs were divided into the following experimental groups (*n *= 4/group). Air: exposed to air and given water to drink; *CS*: exposed to CS and given water to drink; *CS + BT*: exposed to CS and given the BT infusion (2 g/100 ml) to drink instead of water; BT: exposed to air and given the BT infusion to drink. The tea infusion was freshly prepared and replaced every morning and evening. The amount of tea infusion (2% solution) consumed per guinea pig per day was approximately 25 ml (≈0.5 g dry tea).

All guinea pigs were pair-fed individually with respect to a guinea pig in the CS group. The pairs were set up by their initial weights. The amounts of food consumed by the CS group (≈45 ± 5 g/guinea pig/day) was given to the guinea pigs of the other groups. After feeding ascorbate-free diet for 7 days following exposure to CS/air for further 7 days [19], both the sham controls and the CS-exposed guinea pigs were deprived of food overnight and sacrificed next day by diethyl ether inhalation. The lungs were then excised immediately and processed for analysis.

### Histology and morphometric analysis for assessing pulmonary lung damage

The lungs were fixed in 10% formalin and embedded in paraffin. Sections (5 μm) were cut from the periphery of the middle lobes of lungs of each group more or less from similar positions. The paraffin embedded lung tissue sections (5 μm) were deparaffinized using xylene and ethanol (absolute, 95%, 90%, 80%, 70% diluted in water). The slides were washed with phosphate buffered saline (PBS) and permeabilised with permeabilisation solution (0.1 M citrate, 0.1% TritonX-100). The deparaffinized sections were stained with haematoxylin and eosin. Digital images were captured with Olympus CAMEDIA digital camera, Model C-7070 wide zoom (magnification, 10 ×). The individual area (A) and the perimeter (P, the contour length) of each alveolar air space were identified and measured using NIH image. Based on these measurements, a perimeter to area ratio (P/A) was calculated for each alveolar air space. The P/A value was transferred into surface density S/V, using the morphometric relationship S/V = π/4 × P/A [24]. Two images were analyzed per lung section. Altogether 8 images were analyzed in 4 lung sections from each group.

### Oxidative damage of proteins as evidenced by immunoblotting

Oxidative damage of lung proteins was evidenced by immunoblotting of the dinitrophenylhydrazone derivatives of protein carbonyls followed by densitometric scanning as described before [19], with the exception that whole lung lysates were used instead of microsomal membranes.

### TUNEL assay

The paraffin embedded tissue sections (5 μm) were deparaffinized, washed and permeabilised as mentioned above under histology and morphometric analysis. The tunnel reaction was carried out using "In situ cell death detection kit, fluorescein" (Roche) according to manufacture's instruction. After reaction, the slides were washed with PBS and DNA fragmentation was detected by labeling with fluorescein labelled dUTP using terminal deoxynucleotidyl transferase. The cells were examined using a fluorescence microscope (Olympus Bx40) at excitation wavelength of 488 nm. Digital images were captured with cool CCD camera (Olympus; magnification, ×10). The nuclei were counted by counter staining with 4', 6'-diamidino-2-phenylindole (DAPI) at excitation wavelength, 350 nm. Two fields per section of four independent sections in each group were evaluated.

### Immunoblot

The tissue was homogenized in lysis buffer [25]. Protein concentration was measured using Bio-Rad protein estimation kit. Thirty μg of tissue extract was resolved by SDS-PAGE, electro transferred to PVDF membrane, incubated with relevant primary antibodies of recommended dilution, washed and incubated further with HRP conjugated secondary antibodies of recommended dilution and detected using chemiluminiscent kit (Pierce). Caspase 3 and Bax were detected by chemiluminescence kit (Pierce) and Bcl-2 by color reaction using NBT-BCIP reaction. For primary antibodies, anti-rabbit antibodies were used against caspase 3, Bax, Bcl-2, p53, phosphorylated p53 (Ser 392), respectively. In case of tubulin, anti-mouse tubulin antibody was used.

### Statistical analysis

All values are expressed as mean ± SD. Statistical significance was carried out using a two factor ANOVA, with factors being CS and BT. The P values were calculated using appropriate F-tests. Difference with P-values < 0.05 were considered significant.

## Results

### Black tea prevents oxidation of lung proteins of guinea pigs exposed to CS

**Figure 1:**
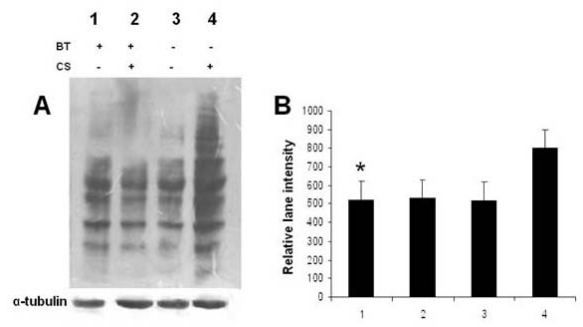
**A**, Oxyblot of lung proteins of guinea pigs exposed to air or cigarette smoke (CS) with or without giving black tea as the drink. The guinea pigs were exposed to air or cigarette smoke (as described under Materials and Methods) and were given water or black tea (BT) as the drink before being sacrificed after 7 days of CS/air exposure. Lane 1, air-exposed guinea pigs given BT as the drink; lane 2, CS-exposed guinea pigs given BT as the drink; lane 3, air-exposed guinea pigs given water as the drink; lane 4, CS-exposed guinea pigs given water as the drink. **B**, Densitometric measurement of the lanes 1, 2, 3, and 4, respectively of Figure 1 **A **using Quantity One- 4.4 (Bio-Rad) Software. * Bars over the respective columns represent means ± SD (n = 4).

Earlier we had reported that CS causes oxidation of guinea pig lung microsomal proteins, which is prevented by BT [19]. Here we show that when guinea pigs are exposed to CS for 7 days and given water as the drink (CS gr), proteins of whole lung tissue are extensively oxidized (Figure 1, lane 4). However, when the CS-exposed guinea pigs are given BT as a drink, CS-induced protein oxidation is completely prevented (Figure 1, lane 2). The immunoblot profile of CS-induced guinea pigs given BT is comparable to those of guinea pigs exposed to air and given water (sham control) or BT as the drink (Figure 1, lanes 1 and 3). The results indicate that oxidation of lung proteins of guinea pigs exposed to CS is prevented by supplementation of BT. Figure 1B represents densitometric measurement of the corresponding lanes of Figure 1A.

### Lung cellular damage in guinea pigs exposed to CS and its prevention by black tea

**Figure 2:**
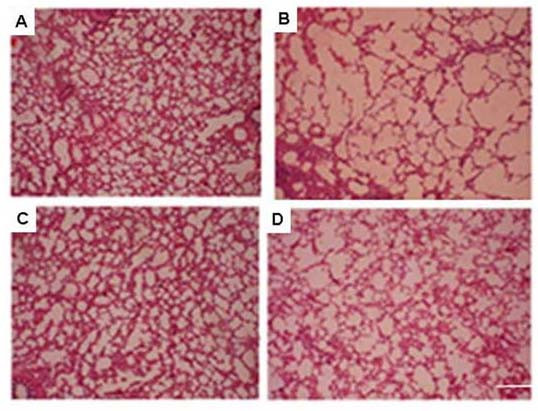
Histopathology profiles of guinea pig lung tissue sections after exposure to air or cigarette smoke in the presence and absence of black tea. Marked enlargement of airspaces was found in lung sections of the guinea pigs in the CS group (see 'Materials and Methods'). The number of guinea pigs used in each group was 4. Eight images were analyzed in 4 lung sections (2 images/section/animal) from each group (magnification ×10). In sharp contrast to the CS-exposed groups (CS group), the enlargement of airspace was greatly reduced in the CS+BT group. The number of air spaces analyzed and the morphometric measurements with statistical difference between the groups are shown in Table 1. **A**, air-exposed guinea pigs given water as the drink (sham control); **B**, CS-exposed guinea pigs given water as the drink; **C**, air-exposed guinea pigs given BT as the drink; **D**, CS-exposed guinea pigs given BT as the drink.

**Figure 3:**
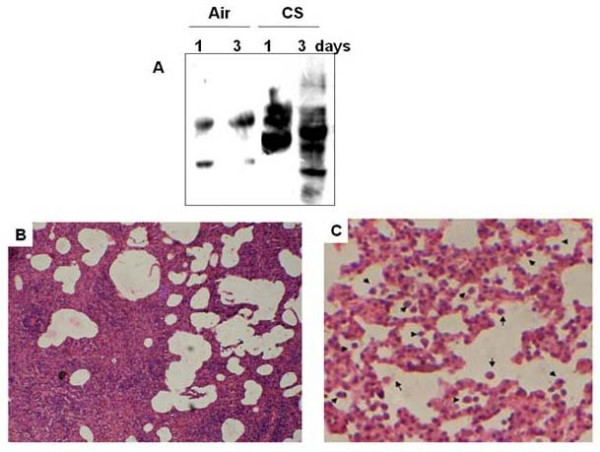
**A**, Immunoblots of the DNP-derivatives of lung proteins of guinea pigs exposed to air or CS after day 1 and day 3. Twenty five μg protein isolated from air-exposed or CS-exposed guinea pigs were converted, without any further treatment, to the DNP-derivative followed by immunoblotting as mentioned in Materials and Methods. 1 and 3 mean exposed to air (sham control) or CS for 1 day and 3 days, respectively. **B, C**, Histopathology profiles of guinea pig lung tissue sections after exposure to cigarette smoke for 3 days. **B **shows infiltration of inflammatory cells in the septal regions. **C **shows accumulation of leukocytes within the alveolar cells that are in all probability macrophages (indicated by → ; magnification × 20)

Histopathology profiles show that when the guinea pigs are exposed to CS for 7 days at an exposure rate of 5 cigarettes (2 puffs/cigarette)/guinea pig/day and given water as the drink, there is marked damage in lung cells, as evidenced by morphometric change and enlargement of airspaces (Figure 2B), as compared to guinea pigs exposed to air and given water as the drink (Figure 2A). When the guinea pigs are exposed to CS and given BT infusion as the drink such change is markedly reduced (Figure 2D). No significant lung cell damage is observed in the guinea pigs exposed to air and given BT as the drink (Figure 2C). Table 1 shows the morphometric measurements of the alveolar air space calculated from 8 different images from each group, including mean area (A) and mean perimeter (P) per image, perimeter per unit area (P/A), and surface density (S/V, surface per unit volume). Actually, the gas exchange (O_2_, CO_2_) of alveolar cells is largely regulated by its surface density [24]. The results show that the S/V value of the CS group (0.155 ± 0.028) is significantly decreased (P < 0.05) from that of the Air group (control, S/V = 0.235 ± 0.038). On the other hand, relative to control BT does not affect the S/V ratio (0.237 ± 0.021, P = 0.851). However, in the presence of CS, BT significantly increases the S/V ratio (0.243 ± 0.029, P < 0.05). The results confirm that compared to CS exposure alone, the increase in alveolar air space is significantly prevented when the CS-exposed guinea pigs are given BT infusion along with smoke exposure. The enlargement of air space is apparently preceded by oxidative protein damage and mild inflammation. Oxidative damage starts on and from the first day of smoke exposure (Figure 3A), whereas inflammation occurs on the third day, as evidenced by infiltration of inflammatory cells in the septal regions and in the alveolar cells (Figures 3B,C,F).

**Table 1:** Morphometric measurements of alveolar air space, perimeter and surface density.

Group	No. of Air Spaces	Total Area (A)*	Total Perimeter (P)*	P/A	S/V
Air	148 ± 10	25576 ± 1754	7646 ± 1282	0.299 ± 0.049	0.235 ± 0.038
CS	84 ± 9	37879 ± 3931	7383 ± 919	0.197 ± 0.036	0.155 ± 0.028
BT	176 ± 12	33978 ± 1704	10239 ± 758	0.302 ± 0.027	0.237 ± 0.021
BT+CS	184 ± 15	35389 ± 2733	10917 ± 1215	0.309 ± 0.037	0.243 ± 0.029

Values are means ± S.D. number of images analyzed in each group:8; A, Total area of air space; P, Total perimeter (contour length) of air space; P/A, perimeter per unit area; S/V (surface density) = π/4 × P/A. The groups represent: Air, air exposed; CS, exposed to CS; BT, air exposed guinea pigs given BT as the drink; CS+BT, CS-exposed guinea pigs given BT as the drink. * Arbitrary unit using NIH image

### Black tea prevents CS- induced apoptosis in the guinea pig lung in vivo

**Figure 4:**
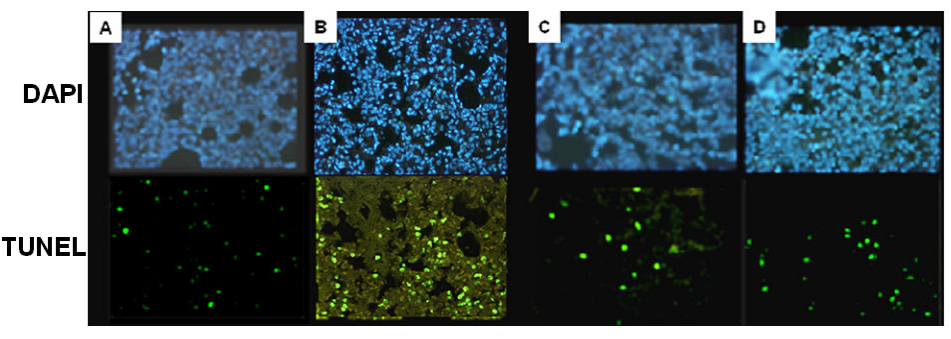
Detection of DNA strand breaks in lung cells of guinea pigs exposed to air or CS in the presence or absence of BT by TUNEL assay. The guinea pigs were exposed to air or CS (as described under Materials and Methods) and sacrificed after 7 days of exposure. Lower Panel: the lung sections were stained with fluorescein labeled dUTP according to the protocols discussed under 'Materials and Methods'. **A**, guinea pigs exposed to air and given water as a drink; **B**, guinea pigs exposed to CS and given water as a drink; **C**, guinea pigs exposed to air and given BT as the drink; **D**, guinea pigs exposed to CS and given BT as the drink. Upper Panel: Lung sections corresponding to the upper panel were counterstained with DAPI to identify the cell nuclei.

**Figure 5:**
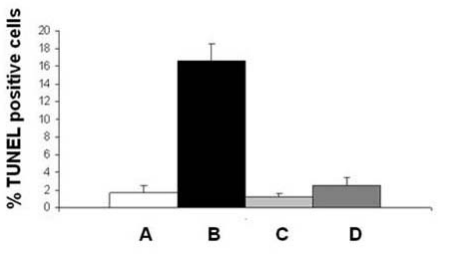
Quantitative evaluation of TUNEL positive cells in lungs of guinea pigs exposed to air or CS in the presence or absence of BT. The percentage of TUNEL positive cells were measured from the results depicted in Figure 4. A,B,C,D, are same as in Figure 4. The number of animals, sections per animal and number of fields analyzed per section were 4, 4 and 2, respectively; the bars over the respective columns represent means ± SD (p < 0.05 between B and A, C or D).

To determine CS-induced apoptosis of alveolar cells *in vivo*, we carried out DNA fragmentation assay (TUNEL assay) on lung sections of guinea pigs of sham control, CS, BT, and CS + BT groups (Figure 4, lower panel). Figure 5 shows that the % of TUNEL positive cells are 1.7 ± 0.9 and 1.2 ± 0.5 (mean ± SD), respectively for air-exposed guinea pigs given water (Figure 5A) or BT (Figure 5C) as the drink. In contrast, marked increase in the TUNEL positive cells (15.7 ± 2.0 SD) is observed in the lung cells of CS-exposed guinea pigs given water as the drink, as indicated by green fluorescence attributable to fluorescein-dUTP labeling (Figure 4B, lower panel and Figure 5B). However, when the CS-exposed guinea pigs were given BT as the drink, the % of TUNEL positive cells decreased to 2.3 ± 1.3 (mean ± SD), which was not significantly different from that of air-exposed guinea pigs (Figure 4D, lower panel and Figure 5D). The results indicate that supplementation of BT prevents CS-induced apoptosis in the guinea pig lung. The upper panel of Fig. 4 shows the nuclei counterstained with DAPI. As observed in the case of inflammation and increased air space, DNA fragmentation is also preceded by oxidative protein damage. While protein oxidation starts from the first day of CS exposure, no significant DNA fragmentation occurs before the third day of smoke exposure.

Quantitative evaluation of the extent of DNA fragmentation indicates that compared to the air-exposed animals, there is 9 fold increase in the TUNEL positive cells in the lung of guinea pigs exposed to CS for 7 days (Figure 5B). However, when the guinea pigs are exposed to CS and given BT as the drink, the % of TUNEL positive cells (Figure 5D) is comparable with that of sham control and the BT group (Figures 5A,C).

### Black tea inhibits CS- induced activation of caspase 3 in vivo

**Figure 6:**
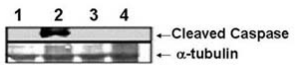
Immunoblot of caspase 3 of the lung extracts of guinea pigs exposed to air or CS given water or BT as the drink. Upper Panel: lane 1, air-exposed guinea pigs given water; lane 2, CS-exposed guinea pigs given water; lane 3, air-exposed guinea pigs given BT; lane 4, CS-exposed guinea pigs given BT. Activation of caspase 3 is evidenced by the formation of cleaved caspase (17 kDa product). Lower panel: the membrane was re-probed with anti-mouse tubulin antibody to determine the level of tubulin as a loading control.

CS induced apoptosis is further supported by checking the level of cleaved caspase 3 by western blotting of lung tissue extract using anti-caspase 3 antibody (Figure 6). The level of cleaved product of caspase 3 (17 KDa) is markedly increased in CS-exposed guinea pigs given water as a drink (Figure 6, lane 2). There is no activation of caspase 3 when CS-exposed guinea pigs are given BT as the drink (Figure 6, lane 4). The level of active caspase 3 is also undetectable in air- exposed guinea pigs given either water (Figure 6, lane1) or black tea as the drink (Figure 6, lane 3).

### Phosphorylation of p53 in the lungs of guinea pigs and its prevention by black tea

**Figure 7:**
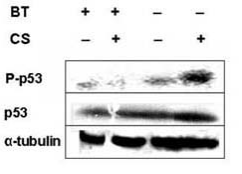
Immunoblot of phosphorylated p53 and p53 of lung extracts of guinea pigs exposed to air or CS given water or BT as the drink. Upper panel represents phosphorylated p53 (P-p53) and lower panel, p53. Lane 1, air-exposed guinea pigs given water; lane 2, CS-exposed guinea pigs given water; lane 3, air-exposed guinea pigs given BT; lane 4, CS-exposed guinea pigs given BT. Details of the experiment are given under 'Materials and Methods'.

Figure 7 shows that the levels of p53 remain unaltered in the lungs of all the groups of guinea pigs, irrespective of whether these are exposed to CS or not. However, the level of phosphorylated p53 is markedly increased in the lungs of guinea pigs exposed to CS and given water as the drink. There is no increase of phosphorylated p53 in the lungs of CS-exposed guinea pigs given BT as the drink (Figure 7).

### Black tea inhibits over expression of Bax in the lungs of guinea pigs exposed to CS

**Figure 8:**
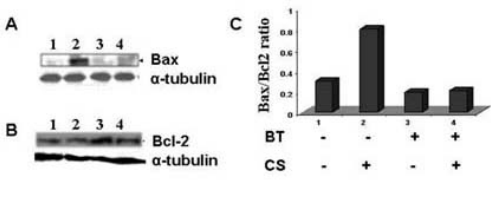
Immunoblot of Bax and Bcl-2 of the lung extracts of guinea pigs exposed to air or CS given water or BT as the drink. Panel A and Panel B depict respective immunoblots of Bax and Bcl-2. Lane 1, air-exposed guinea pigs given water; lane 2, CS-exposed guinea pigs given water; lane 3, air-exposed guinea pigs given BT; lane 4, CS-exposed guinea pigs given BT. In each case the membrane was reprobed with anti-mouse tubulin antibody to determine the level of tubulin as a loading control. Panel C shows the Bax/Bcl-2 ratio observed in different groups.

It is known that one mechanism of apoptosis is over expression of Bax, a member of the Bcl-2 family. Figure 8A (lane 2) shows that the level of Bax protein increased significantly (p < 0.05) in lung extract of guinea pigs exposed to CS and given water as the drink. In contrast to this, when CS-exposed guinea pigs were given BT as the drink, there was no over expression of Bax (Figure 8A, lane 4). Also there was no increase of Bax in the lungs of guinea pigs exposed to air and given either water (Figure 8A lane1) or BT (Figure 8A, lane 3) as the drink. It is known that while Bax is proapoptotic, Bcl-2, is antiapoptotic. We therefore examined the level of Bcl-2 proteins. While the level of Bax protein significantly increased in response to CS treatment given water as the drink (Figure 8A, lane 2), CS did not affect the level of Bcl-2 proteins (Figure 8B, lane 2). This resulted in an increase in the ratio of Bax/Bcl-2, as measured by densitometric scanning (Figure 8C). These observations suggest that the apoptotic effect of CS on guinea pig lung cells was caused by an increase of Bax/Bcl-2 ratio. Although there was some increase in the Bcl-2 level in CS-exposed guinea pigs given BT as the drink (Figure 8B, lane 3), the ratio of Bax/Bcl-2 did not increase (Figure 8C, column 3).

## Discussion

We had previously demonstrated that cigarette smoke causes oxidation of guinea pig lung microsomal proteins [14,15,19]. Here we demonstrate that exposure of marginal vitamin C-deficient guinea pigs to CS causes oxidation of whole lung proteins. We have used vitamin C-depleted guinea pigs to minimize the ascorbate level in the tissues. This is because ascorbate is a potential inhibitor of CS-induced oxidative protein damage [14,15]. If the guinea pigs were fed ascorbate-rich diet (15 mg vitamin C/animal/day), the animals failed to respond to CS [15]. We further demonstrate that oxidative modification of proteins by cigarette smoke leads to inflammation, apoptosis and cellular damage of the lung (increased air space) and that black tea can prevent such cigarette smoke-induced lung damage. Others had also shown that one major deleterious effect of smoking is oxidative damage of proteins [13,16]. Such oxidative modifications of structural proteins in the lung, including protein carbonylation, play a significant role in the etiology and progression of several human pulmonary diseases [13,26,29]. Oxidative stress plays an important role not only through direct injurious effects, but by involvement in the molecular mechanisms that control lung inflammation (13). One intriguing aspect of such oxidative protein damage is that the oxidized proteins become vulnerable to degradation by endogenous proteases present in the tissues [14,29-32]. This may be a key cause of the degradation of lung structural proteins in smokers leading to degenerative diseases like emphysema, which is marked by the loss of structural matrix of the lung and its elasticity leading to impaired transfer of oxygen and carbon dioxide into and out of the blood. We have shown that protein oxidation is followed by inflammation, as evidenced by infiltration of inflammatory cells in the septal region and macrophages inside the alveoli. It is known that during phagocytosis macrophages undergo oxidative burst, accompanied by release of proteases [33]. The proteases released from activated macrophages along with the endogenous proteases present in the tissue may be involved in degrading the cytoskeletal proteins leading to destruction of alveolar membranes and septal cells in emphysema. It is thus conceivable that if oxidation of lung proteins is prevented by antioxidants, subsequent proteolysis would be prevented, and this in turn would prevent lung damage like that observed in emphysema. Here we show that oxidation of proteins (Figure 1) and accompanied damage to the lung cells (Figure 2) are both inhibited by giving the CS-exposed guinea pigs BT as the drink. The extent of lung damage by CS exposure has been evidenced by the significant increase of the surface density (S/V) of the alveolar air space (Table 1). This represents the membrane interface of each alveolar air space per unit area. It is known that the efficiency of gas exchange (O_2 _and CO_2_) is greatly regulated by the surface density [24]. The S/V is significantly increased by giving the CS-exposed guinea pigs BT as the drink. The possibility that the loss of alveoli accompanied by increased air space in the CS-exposed guinea pigs was due to inanition and comparatively less calorie intake [34] is not tenable. This is because the CS-exposed guinea pigs consumed ≈45 ± 5 g diet/day and the guinea pigs of all other groups, namely, sham control (air-exposed), BT, and CS + BT group were pair-fed with respect to the CS group. We had shown before that the inhibitory effect of BT is apparently a synergistic effect of the antioxidant flavonols present in BT, namely, theaflavins (TF), thearubigins (TR) and catechins (CT) [19]. Based on the flavonol contents of BT, as determined before [19], the amount of flavonols consumed per guinea pig per day was approximately 5 mg TF, 90 mg TR and 30 mg CT. The BT flavonols probably act by quenching the stable oxidants, which might be long-lived radicals present in CS that are apparently responsible for oxidation of the lung proteins [14,35,36].

Our present data and other reports indicate that along with oxidative damage, apoptosis plays a crucial role in CS-induced lung damage [1,4-8]. Although it is hypothesized that interaction of oxidative stress and apoptosis leads to pathophysiological conditions in emphysema, the question remains to be addressed: which one is the initial event, oxidative damage or apoptosis? It has been proposed that a vicious cycle may be established, because cells undergoing apoptosis display increased oxidative stress, which further contributes to the apoptosis [5]. The role of apoptosis in such lung damage is not mere correlative, but potentially causative [6]. Here we show that the oxidative damage is the initial event, which is followed by inflammation, apoptosis and increased air space indicating emphysematous change. The biochemical events that mark such apoptotic changes are DNA fragmentation, over-expression of Bax and activation of caspase 3. We have demonstrated that marked DNA fragmentation (increase in TUNEL positive cells) occurs in lungs of CS-exposed guinea pigs given water as the drink (Figures 4 and 5). When the CS-exposed guinea pigs are given BT instead of water, there is no observable increase in the DNA fragmentation. The percentage of TUNEL positive cells are comparable to that of sham controls (Figures 4 and 5). This indicates that BT prevents CS-induced DNA fragmentation.

Aoshiba et al. [10] reported that acute cigarette smoke exposure induces apoptosis of alveolar macrophages. However, Aoshiba et al. worked with rats and the present authors with partially vitamin C-deprived guinea pigs. Also, in the present study the authors used a relatively mild challenge while that used by Aoshiba et al. [10] was more severe, as evidenced by occurrence of some degree of alveolar bleeding. This is never observed in human smokers. Moreover, the incidence of alveolar macrophage (AM) apoptosis in CS-exposed rats obtained by Aoshiba et al. [10] was much lower (3.2 %) than observed by the present authors (≈16 %). This difference might be due to the fact that rats synthesize vitamin C [21] and vitamin C present in the respiratory tract of rats might have prevented the effect of CS inhalation on AM apoptosis.

Caspases contribute to apoptosis through disassembly of cell structures by disrupting the nuclear structure and also by cleaving several cytoskeletal proteins [30,37]. Caspases are synthesized initially as inactive single polypeptide chains that undergo proteolytic cleavage to produce subunits having active protease activity. We have shown in this report that CS causes cleavage of procaspase 3 to active caspase 3 (17 KDa, Figure 6) in the guinea pig lung. When the CS-exposed guinea pigs were given BT as a drink, activation of caspase 3 was prevented (Figure 6).

It has already been demonstrated that phosphorylated form of p53 accumulates in the nucleus in response to DNA damage [38]. We have shown here that although the level of p53 in the guinea pig lung remains unaltered after exposure to CS, the level of phosphorylated p53 is markedly increased (Figure 7). Phosphorylation of p53 and its trnslocation in the nucleus is accompanied by expression of Bax. Here we show that besides preventing CS-induced oxidation and fragmentation of DNA, BT also prevents CS-induced phosphorylation of p53 (Figure 7). In fact, we observed practically no accumulation of phosphorylated p53 in the lungs of guinea pigs exposed to CS and given BT as the drink (Figure 7).

Apoptosis is regulated by expression of a number of genes, including the Bcl-2 family [39,40]. Out of these Bax is pro-apoptotic and Bcl-2 is anti-apoptotic. So, the ratio of Bax and Bcl-2 determines whether a cell will undergo apoptotic death or not. We have shown that CS exposure to guinea pigs given water as the drink has no effect on the level of Bcl-2, whereas the Bax protein is significantly increased, resulting in an overall increase of Bax/Bcl-2 ratio (Figure 8C, column 2). When the CS-exposed guinea pigs were given BT as the drink, there was no over expression of Bax (Figure 8A, column 4). This resulted in a reversal of the Bax/Bcl-2 ratio (Figure 8C, column 4). Although densitometric measurement shows that there is an increase of Bcl-2 proteins in the presence of BT (25% in lane 3 and 13% in lane B, over that of lanes 1 and 2, Fig 8), the significance of this increase is not clear.

In conclusion, we demonstrate that there is a close link between oxidative damage, apoptosis and lung cellular damage in our guinea pig model exposed to cigarette smoke. Apparently, the initial event in the pathophysiological condition is oxidative damage of proteins. This is followed by inflammation and apoptosis leading to destruction of alveolar membranes and septal cells, resulting in increased air space in the lung. When the CS-exposed guinea pigs are given BT as the drink, oxidative damage is prevented and this is accompanied by the prevention of apoptosis and lung damage.

The present study has some limitation for consideration of the smoke-induced guinea model as a model of COPD. In human smokers with COPD, marked inflammation associated with massive neutrophil influx is often seen. However, neutrophil accumulation is not a feature of the present model. Nevertheless, besides inflammation and neutrophil influx the CS-induced lung damage produced in guinea pigs may be comparable to that of human smokers. The structure of the guinea pig lung has similarity with that of the human lung with three major lobes on the right and two major lobes on the left as well as well-defined terminal bronchiole with subtending alveolar ducts (41). Also, the guinea pig develops morphologic and physiologic alterations after exposure to CS at the same pattern as humans [41]. So the results obtained with guinea pigs in our present study would imply that regular intake of black tea may protect smokers from the risk of developing lung damage.

## Abbreviations

CS, cigarette smoke; BT, black tea; Control, exposed to air and given water as the drink (also called Air group in Table 1); CS gr., exposed to CS and given water to drink; CS + BT gr., exposed to CS and given BT infusion as the drink; BT gr., exposed to air and given BT infusion as the drink.

## Competing interests

The author(s) declare that they have no competing interests.

## Authors' contributions

SB designed the experiments and carried out majority of the work. IBC planned the experiments and wrote the manuscript, including revision. KP, SM and DJ participated in the study of oxidative damage. AKS and PM participated in the study on signaling. All authors read and approved the final manuscript.
